# Investigation of Delamination Characteristics in 3D-Printed Hybrid Curved Composite Beams

**DOI:** 10.3390/polym16162250

**Published:** 2024-08-08

**Authors:** Sedat Süsler, Zafer Kazancı

**Affiliations:** 1 Faculty of Aeronautics and Astronautics, Kocaeli University, Kartepe 41285, Kocaeli, Turkey; sedat.susler@kocaeli.edu.tr; 2 Advanced Composites Research Group, School of Mechanical and Aerospace Engineering, Queen’s University Belfast, Belfast BT9 5AH, UK

**Keywords:** curved beam, delamination, continuous fibre, additive manufacturing, continuous filament fabrication, hybrid composite

## Abstract

This study focuses on understanding the impact of different material compositions and printing parameters on the structural integrity of hybrid curved composite beams. Using the continuous filament fabrication technique, which is an advanced fused deposition modelling process, composite curved beams made of short carbon and various continuous fibre-reinforced nylon laminae were fabricated and subjected to four-point bending tests to assess their delamination characteristics. The results show that the presence of five flat zones in the curved region of a curved beam achieves 10% and 6% increases in maximum load and delamination strength, respectively, against a smooth curved region. The delamination response of a curved composite beam design consisting of unidirectional carbon/nylon laminae is superior to that of a curved beam made of glass fibre/nylon laminae, while the existence of highly strengthened glass fibre bundles is alternatively quite competitive. Doubling the number of continuous fibre-reinforced laminae results in an increase of up to 36% in strength by achieving a total increase in the beam thickness of 50%, although increases in mass and material cost are serious concerns. The hybrid curved beam design has a decrease in the maximum load and the strength by 11% and 13%, respectively, when compared with a non-hybrid design, which consists of some type of stronger and stiffer nylon laminae instead of short carbon fibre-reinforced conventional nylon laminae. Two-dimensional surface-based cohesive finite element models, which have a good agreement with experimental results, were also established for searching for the availability of useful virtual testing. The results from this study will greatly contribute to the design and numerical modelling of additively manufactured hybrid composite curved beams, brackets, and fittings.

## 1. Introduction

Fused deposition modelling (FDM), which is one of the most popular additive manufacturing (AM) technologies used today, has been advancing greatly in manufacturing 3D-printed complex composite structures. It has gained significant attention across next-generation applications in the aerospace and automotive industries [1]. Researchers and engineers continue to explore and refine the capabilities of this technology, pushing the boundaries of what is possible in the world of advanced materials and manufacturing. FDM harnesses the layer-by-layer deposition capability of 3D printing alongside the versatility of polymer composite materials, which consist of a polymer matrix and reinforcing components. By combining these elements, 3D printing with composites enables the creation of intricate structures with customised properties. This innovative method facilitates precise customisation, swift prototyping, iterative design, waste reduction, cost efficiency, and user-friendly operation, catering to diverse lightweight industrial needs.

Conventionally moulding-based manufactured composite brackets, which are feasible for printing via FDM due to their complex geometry too, are well on the way to attaining common usage and taking over from metallic ones, especially in the aerospace industry. The contributions of more studies on composite curved (L-shaped) beams, which lead to better delamination performance, are the milestones to designing more reliable composite brackets since they can be defined in the form of a simplified bracket with a single bend. A 90° bend is the most critical region of a curved beam under operational loading conditions. Designing and printing composite curved beams using continuous fibres via continuous filament fabrication (CFF), which is an advanced FDM process, is a recent method that is competitive with conventional composite manufacturing methods, although the attempt is still in its infancy [2]. Most of the mechanical properties of additively manufactured continuous fibre-reinforced non-hybrid and hybrid composites were studied and reviewed by also considering the effects of printing parameters [3,4,5,6]. However, studies on the damage mechanism and strength of 3D-printed composite curved beams using continuous fibres are limited to curved parts made of just a unidirectional (UD) carbon fibre bundle reinforced nylon lamina and their damage mechanism based on the fibre buckling and breakage [7,8,9]. Moreover, a fibre polymer laminate (FPL) design for 3D-printed composite curved beams, which was inspired by the concept of fibre metal laminates (FMLs), was proposed in the form of a laminate made of five strengthened nylon materials (Nylon White) and two continuous UD fibre-reinforced nylon laminae by Süsler and Kazancı [2]. Delamination strength comparisons of composite curved beams made of various continuous fibre materials such as carbon, glass, high-strength high-temperature glass, and Kevlar^®^ subjected to four-point bending were analysed experimentally and numerically. The effect of the number of curved beams per build during multiple printing was also included in that study. To the author’s knowledge, there is no research on the delamination behaviours of hybrid laminated composite curved beams considering print resolution, continuous fibre material, and thickness effects.

A damage mechanism of polymer matrix composite materials based on delamination occurs and can be analysed if interlaminar shear stress exceeds interlaminar shear strength (ILSS) of a short-flat or a short-curved beam subjected to three-point bending via the standardisation of ASTM D2344/D2344M-22 [10] and interlaminar tensile stress exceeds interlaminar tensile strength (ILTS) of a curved beam with long flat legs under four-point bending loading via the standardisation of ASTM D6415/D6415M-22 [11]. There are some experimental and numerical studies that have extensive analyses of the delamination performance and ILSS of 3D-printed continuous fibre-reinforced short flat beams [12,13,14,15,16]. On the other hand, some authors experimentally focused on the Mode I and Mode II interlaminar fracture toughness properties of various additively manufactured composite materials made of continuous fibres [16,17,18,19,20].

In this present study, the curved beams subjected to four-point bending loading are designed and printed as hybrid FPLs, which are a combination of short carbon fibre-reinforced nylon (Onyx) and continuous UD fibre-reinforced nylon laminae. The existence of flat zones in the curved region on the delamination strength is evaluated. The comparison between certain curved beams made of different continuous fibre materials such as carbon, glass, and high-strength, high-temperature glass is made. Another material effect is examined by comparing the existence of Onyx lamina in the beam with the non-hybrid design in Ref. [2]. The effect of the thicker beam, which leads to an increase in the number of embedded fibre bundles for all types of continuous fibre-reinforced beams, on the delamination strength is taken into account in the next step. Load-displacement histories are obtained experimentally. Curved beam moment per unit width-displacement and maximum interlaminar tensile stress (MILTS)-displacement histories, whose peak values are respectively CBS and ILTS, are calculated analytically by using experimental data to observe the relation between the change in values and the damage mechanism during a test. Failure modes and radial failure locations of curved beams are examined as well. Two-dimensional surface-based cohesive numerical models of the selected 3D-printed composite curved beams are also analysed, and the outputs are compared with the experimental results.

## 2. Materials and Methods

### 2.1. Composite Curved Beam and AM Method

**Figure 1:**
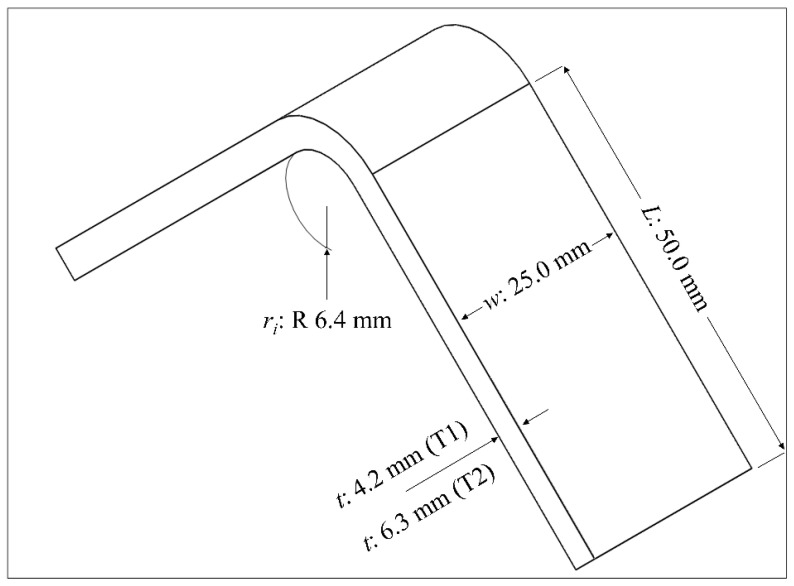
The basic geometry of the curved beam.

The basic dimensional configuration of the curved beam depended on the ASTM standard [11], which suggests an L-shaped design. The solid beam contained two straight loading legs, which were joined at a 90° angle, and a curved region with a 6.4 mm inner radius (*r_i_*) between the two legs. The entire beam had a 25 mm width (*w*). The length of each straight leg (*L*) was assigned as 50 mm. The outer radius (*r*_0_) of the curved region changed according to the thickness (*t*), which was selected as 4.2 mm (denoted by T1) and 6.3 mm (denoted by T2). The basic dimensions are demonstrated in the scheme in Figure 1.

**Figure 2:**
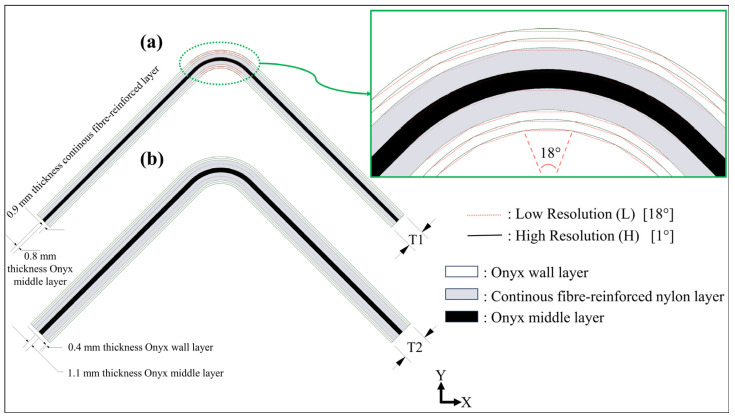
The layouts of certain curved beam designs: (**a**) 4.2 mm-thickness curved beams with low and high-resolution options; (**b**) 6.3 mm-thickness curved beam.

A short carbon fibre-reinforced nylon material called Onyx, which contains an approximate 130 µm carbon fibre length and has a 14% mass fraction of short carbon [21], and reinforced nylon materials, which contain various continuous fibres such as carbon, glass, and high-strength high-temperature glass, were used to print certain hybrid curved beam configurations as FPL designs. Figure 2a shows T1-thickness curved beam layouts in detail, whose curved region consisted of five flat zones or whose curved region was smooth in the *XY* plane. Figure 2b shows a T2-thickness curved beam layout, which was designed to have a smooth curved region only. The beam with five flat-zoned curved regions was defined as a low-resolution design, which was set by an angular tolerance of 18° while creating an STL print file. On the other hand, the high-resolution design created a perfectly regular arc consisting of ninety flat zones in the curved region via the selection of an angular tolerance of 1°.

The T1-thickness laminate had two Onyx wall layers (0.4 mm thickness for each), a continuous UD fibre-reinforced nylon layer (~0.9 mm), an Onyx middle layer (~0.8 mm), a UD fibre-reinforced nylon layer, and two Onyx wall layers from the top to the bottom, respectively. The T2-thickness laminate had the same upper and lower wall layers as the thinner laminate, while it was reinforced with two UD fibre-reinforced nylon layers positioned on each side of an Onyx middle layer (~1.1 mm). Continuous carbon, glass fibre, and high-strength, high-temperature glass fibre-reinforced nylon layers were formed during the printing step, in which a special thermoplastic prepreg bundle with a diameter of 0.3 mm melted after the filament had moved from the spool through the fibre nozzle at 250 °C. Onyx layers were printed at 275 °C separately after Onyx filament had moved from its spool to the plastic nozzle and had melted.

After the basic geometry of curved beams had been designed via Solidworks^®^ [22] and STL print files had been created, the composite laminates were arranged using Eiger slicing software [23], as stated by the printing inputs in Table 1. The parts were then printed by a Markforged Mark Two printer [24]. The warping problem during printing on the thin edges of curved beams was solved by adding brims and rafts that surround and support the bases of specimens on the print bed. The brim and the raft of a specimen were then easily removed before the test. The print configuration was optimally chosen as an on-edge orientation that can allow the embedding of continuous UD fibre bundles into the curved region [2].

The printer achieves 0.125 mm print layer thickness in the *Z* direction for continuous carbon-reinforced structures, unlike glass fibre and strengthened glass fibre-reinforced structures, which have a 0.1 mm thickness each. The other limitation in the *Z* direction is the necessity to add floor and roof Onyx layers, which are five each, for covering up the fibre-reinforced region in the *Z* direction. As a result, the 25 mm *w*-curved beam was formed by continuous carbon-reinforced nylon cross sections along 23.75 mm positioned in the middle, plus 0.625 mm Onyx floor and roof sections. Glass fibre and high-strength, high-temperature glass fibre-reinforced nylon cross sections were similarly positioned along 24 mm in *w* by including 0.5 mm Onyx floor and roof sections to form the 25 mm *w*-curved beams.

**Figure 3:**
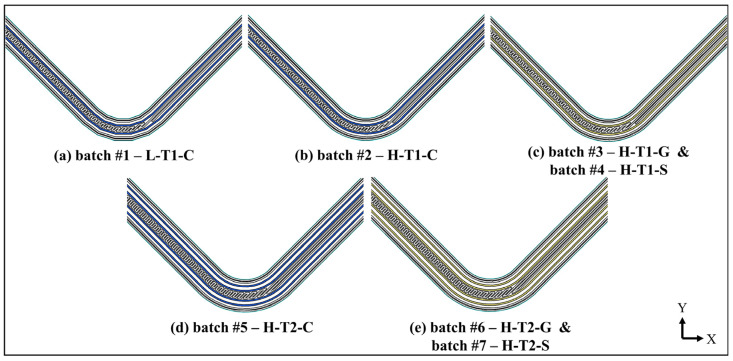
Batches of specimens with their codes and the views of their 2D build orientations on the print bed: (**a**) batch 1, (**b**) batch 2, (**c**) batch 3 and batch 4, (**d**) batch 5, (**e**) batch 6 and batch 7.

Seven batches of specimens were printed and encoded according to their curved regions’ resolutions, continuous fibre materials and total thicknesses. The batches and their codes are shown with their 2D and 3D build orientations in Figure 3. The sub-codes are shown with their descriptions for a better understanding in Table 2. For example, the batch 1’s code, L-T1-C, denotes a 4.2 mm thickness-curved beam, which contains flat portions in its curved region, and which is made of continuous carbon fibre-reinforced nylon laminae.

When *υ_m_* is the volume of Onyx used for each specimen of certain batches during printing and *υ_f_* is the volume of the continuous fibre-reinforced nylon, *V_r_*, which can be defined as the continuous fibre-reinforced region volume ratio, is calculated as the ratio of *υ_f_* to (*υ_m_* + *υ_f_*) by excluding the volume of Onyx for brim and raft and neglecting the void content. These values are summarised for each batch of specimens in Table 3. Moreover, the predicted final part mass and material cost of a specimen of each batch were obtained via Eiger software similarly and are shown in Table 3. Having a smooth, curved region instead of flat portions did not affect the printing outputs. Embedding strengthened glass bundles instead of conventional glass fibres had no change on printing outputs except for a cost increase of up to 21%. Doubling the number of continuous fibre-reinforced laminae resulted in an increase of up to 53%, 86%, and 50% in final part mass, material cost, and *V_r_*, respectively.

**Figure 4:**
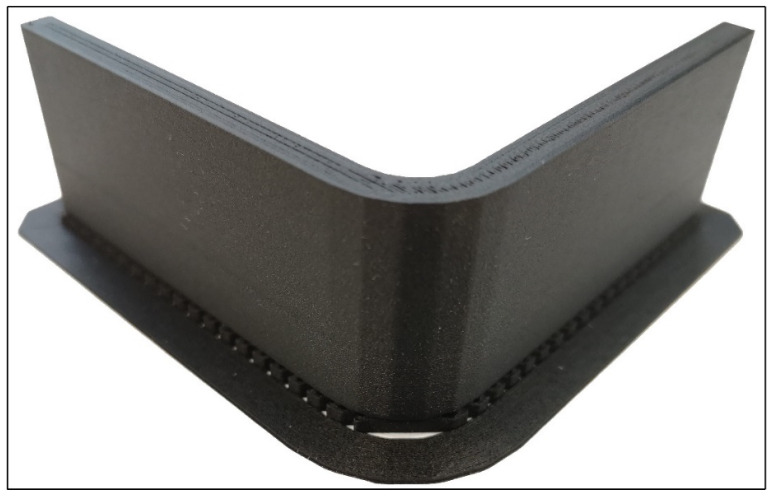
A finished five-flat zoned L-T1-C product on its brim and raft.

**Figure 5:**
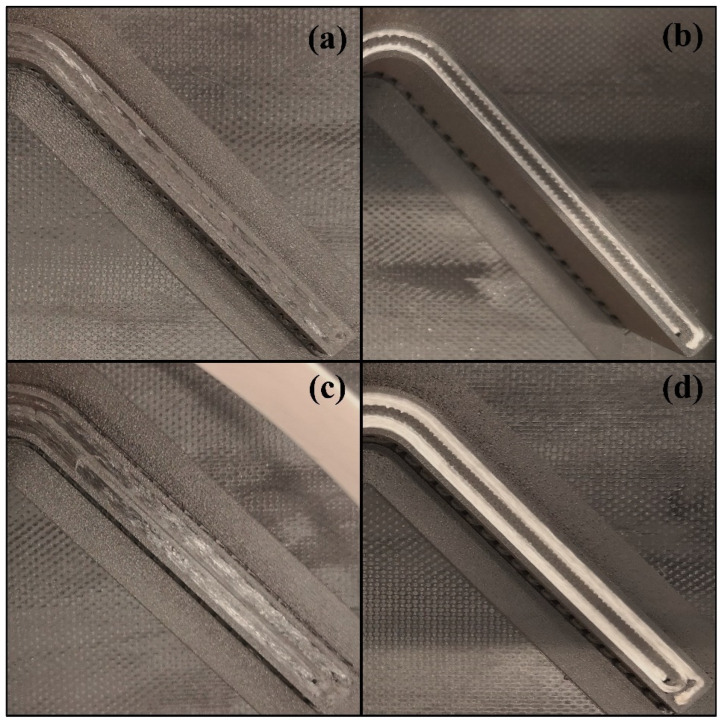
The top views of cross sections: a specimen of (**a**) H-T1-C, (**b**) H-T1-G, (**c**) H-T2-C, and (**d**) H-T2-G.

Figure 4 shows a finished specimen of the L-T1-C batch on its brim and raft. Five flat zones are clearly noticed in the curved region. Top views of the cross sections of high-resolution curved beams are shown and compared with each other in Figure 5. It is approved that the cross sections of the FPL designs in Figure 2 cohere with the printed ones.

### 2.2. Test Method

When a UD fibre-reinforced composite curved beam is subjected to bending load via a proper four-point bending apparatus, a sufficient curved beam moment, which creates interlaminar tensile stress through the thickness of the curved region, occurs and is defined as follows:

(1) $$M=P_{b}l_{0}$$

where *P_b_* is the force applied to one of the supporting bars, and *l*_0_ is the straight distance between that supporting bar and the loading bar. The curved beam moment per unit width is expressed as CBS at the start of initial delamination, and it can be defined as follows:

(2) $$\frac{M}{w}=\left(\frac{P}{2w\cos\varphi }\right)\left(\frac{l_{b}-l_{t}}{2\cos\varphi }+\left(D+t\right)\tan\varphi \right)$$

where *P* defines the force applied to the apparatus. *φ* is the angle between one of the specimen legs and the horizontal axis during a test. *l_b_* expresses the lower span length, while *l_t_* is the upper span length, and *D* is the diameter of the cylindrical bar. *φ* is 45° when *P* is 0, and it is critical to calculate its actual value when CBS is obtained since *φ* decreases during a test. If Δ is the crosshead displacement of the test machine at a certain *P*, *φ* is expressed as follows:

(3) $$\varphi =\sin^{-1}\left(\frac{-d_{x}\left(D+t\right)+d_{y}\sqrt{d_{x}^{2}+d_{y}^{2}-D^{2}-2Dt-t^{2}}}{d_{x}^{2}+d_{y}^{2}}\right)$$

where

(4) $$d_{x}=\frac{l_{b}-l_{t}}{2}\;\;\;;\;\;\;d_{y}=d_{x}+\frac{2\left(D+t\right)}{\sqrt{2}}-\Delta$$

The standard ASTM D6415/D6415M-22 [11] suggests using Equation (5) for the prediction of ILTS of a UD fibre-reinforced polymer matrix composite, which has a ratio of longitudinal Young modulus (*E*_1_) to transverse Young modulus (*E*_2_) less than 20 since an error of maximum 2% is produced. The error obtained was fewer (less than 0.5%) for 3D-printed composites before [2].

(5) $$ILTS=\frac{3\cdot CBS}{2t\sqrt{r_{i}r_{0}}}$$

**Figure 6:**
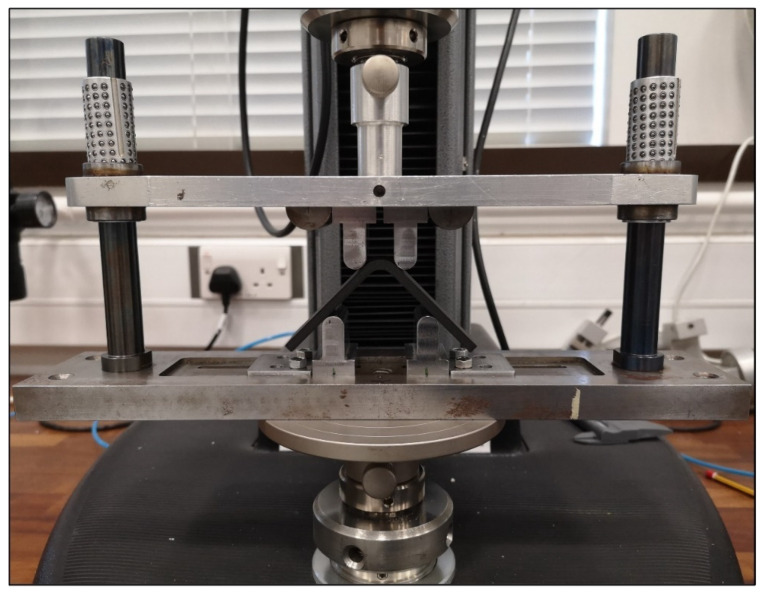
The experimental setup.

The tests were carried out using a four-point bending fixture that was mounted between the grips of the Lloyd LS5 tensile/compression machine [25], as shown in Figure 6. The lower part of the fixture was fixed while the upper part was moving downward at a crosshead speed of 3 mm/min. *D* of each loading and supporting cylindrical steel bar was 10 mm. *l_b_* and *l_t_* were arranged as 42.1 mm and 22.1 mm, respectively. The 90° angle between the beam legs, the 18° angle of flat zones in the curved regions of the L-T1-C batch, the *t* values, which were T1 and T2, and the 25 mm *w* were measured before tests, and the sensitive manufacturing capability of AM technology was confirmed for all samples.

*P* and Δ data were collected during each test at a sampling rate of 10 Hz. Calculations of curved beam moment per unit width and MILTS were made for every obtained *P* and Δ. CBS and ILTS values were obtained for each batch. However, the *w* of Onyx floor and roof layers were neglected and were excluded from the total *w* in Equation (2). Their contribution to delamination strength is so limited by comparison with continuous fibre-reinforced sections according to 3D finite element analysis (FEA) with solid elements [2]. *w* was consequently defined as 23.75 mm, 24 mm, and 24 mm for the batches of C, G, and S, respectively. A test matrix in Table 4 summarises the batch properties and the experimental campaign performed.

### 2.3. Finite Element Modelling

FEA was used for modelling the problem in this study, although there are other powerful tools to model a composite structure and do a stress analysis like the finite difference method [26] and the Bézier-based multi-step method [27]. The curved beam batches, L-T1-C, H-T1-C, and H-T2-C, were modelled in 2D planar via commercial Simulia Abaqus/CAE 2021finite element software [28]. The deformable 4.2 mm and 6.3 mm thickness cross sections in Figure 2 consisted of separately modelled seven and nine laminae, respectively, in total, while each 2D cylindrical roller was defined as the discrete rigid wire. Adjacent two wall layers in all finite element models and two adjacent carbon/nylon laminae in the H-T2-C model were perfectly bonded to each other. A surface-based bilinear traction-separation cohesive contact, which has well-generated cohesive properties, was defined between a continuous UD carbon/nylon and Onyx laminae.

**Figure 7:**
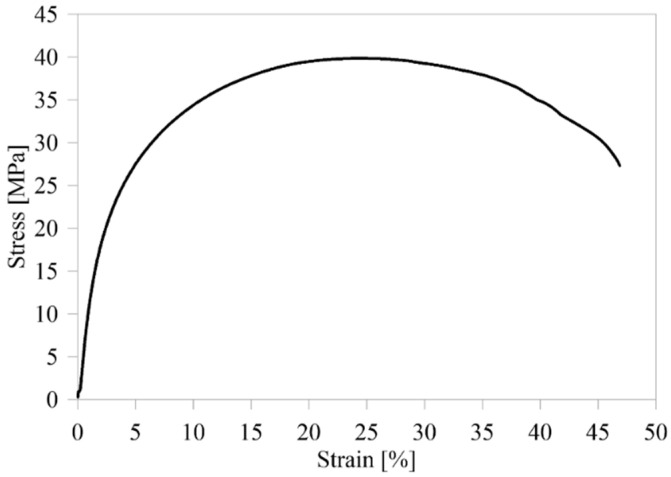
Tensile stress-strain curve of Onyx [21].

In-plane elastic constants of a 3D-printed UD carbon/nylon lamina, which had been obtained by an experimentally validated numerical study by Galati et al. [29], were used in the finite element model. Moreover, the material properties, including the ultimate tensile stress (UTS) and stress-strain curve of Onyx, which had been printed in on-edge orientation and had been tested at a crosshead speed of 5 mm/min by Fisher et al. [21], were used. Table 5 shows the material properties of the laminae used in this study. The stress-strain curve of the Onyx specimen, which has the UTS closest to the mean one, is plotted in Figure 7 in order to include plastic behaviour and ductile damage to the Onyx material in FEA.

Material properties were assigned to 2D models as sections. Material orientations in two directions were arranged to form a UD laminated structure in which all continuous fibres run continuously through the legs and the curved region of the beams. A contact property between the surfaces of the beam sections and the 2D rigid rollers, which had both normal hard contact and frictionless tangential behaviour, was created according to the configuration of the virtual test setup. The clamped boundary condition was defined for the lower rollers, while the upper rollers are only allowed to have a certain displacement in the Y direction.

**Figure 8:**
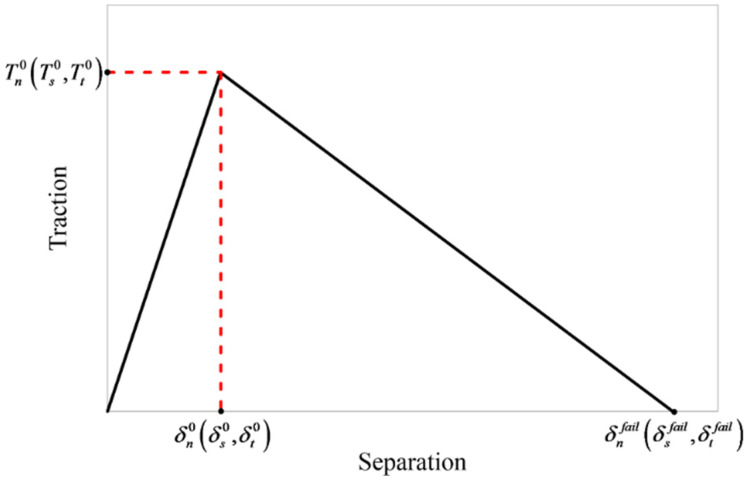
Traction-separation response of a cohesive contact.

The simulated cohesive behaviour was acted upon by a zero-thickness cohesive zone that used surface interaction properties unlike the material properties of conventional cohesive elements. It was consistent to use a surface-based cohesive contact pair in this study, as the interface thickness between a printed carbon/nylon lamina and an Onyx lamina was negligibly small in comparison with a conventional adhesion. A common traction-separation response is shown in Figure 8. The normal and shear traction stresses (*T_n_*, *T_s_*, *T_t_*) increase linearly until reaching separation vectors (*δ_n_*^0^, *δ_s_*^0^, *δ_t_*^0^) at peak strength points (*T_n_*^0^, *T_s_*^0^, *T_t_*^0^) in the first part of the response, which represents an elastic behaviour.

*T_n_*, *T_s_,* and *T_t_* are generated in the first part of the response as follows:

(6) $$\left[T_{i}\right]=\left[\begin{array}{c} T_{n}\\ T_{s}\\ T_{t} \end{array}\right]=\left[k_{ii}\delta_{i}\right];\;\;\;i:n,s,t$$

where the penalties are only normal (*k_nn_*) and shear stiffness components (*k_ss_*; *k_tt_*) in an uncoupled model. The traction stresses are proportional to the separation vectors, which are *δ_n_*, *δ_s_,* and *δ_t_*. Delamination initiation in the cohesive contact is activated when defined damage initiation criteria, which were selected as a quadratic stress criterion:

(7) $${\left(\frac{T_{n}}{T_{n}^{0}}\right)}^{2}+{\left(\frac{T_{s}}{T_{s}^{0}}\right)}^{2}+{\left(\frac{T_{t}}{T_{t}^{0}}\right)}^{2}=1$$

A damage variable (*d*), which is defined from 0 to 1, generates the propagation model of the damage in the second part of the response in Figure 8:

(8) $$\left[T_{i}\right]=(1-d)\left[k_{ii}\delta_{i}\right]\;\;;\;\;\;i:n,s,t$$

The area under the traction-separation curve defines the dissipated fracture energy. An energy-based mixed mode approach is consequently effective in simulating damage propagation via the Benzeggagh and Kenane (BK) fracture criterion [30] when *G_s_^C^* = *G_t_^C^* is assumed:

(9) $$G^{C}=G_{n}^{C}+\left(G_{s}^{C}-G_{n}^{C}\right){\left(\frac{G_{S}}{G_{T}}\right)}^{m}$$

where *G_n_^C^*, *G_s_^C^,* and *G_t_^C^* are the critical normal, first shear, and second shear energy release rates, respectively. *G_S_* is the sum of shear fracture energies, *G_T_* is the sum of normal and shear fracture energies, and *m* is the BK cohesive parameter.

*k_nn_*, *k_ss_,* and *k_tt_* are simply predicted in Equation (10) when it is assumed that all stiffness components are the same. *α* is a parameter that is overly bigger than 1 (50 is recommended), and each sub-laminate has the thickness *t*, while the out-of-plane Young modulus is *E*_3_ [31].

(10) $$k_{nn}=k_{ss}=k_{tt}=\frac{\alpha E_{3}}{t}$$

where *t* was defined as the mean value of a UD carbon/nylon and an Onyx lamina and was selected as 0.85 mm, 0.85 mm, and 0.9 mm for the finite element models of the batches L-T1, H-T1, and H-T2 of C type, respectively. *E*_3_ was obtained by using the Halpin-Tsai semi-empirical equation for *E*_2_ [32], when the beam made of carbon/nylon and Onyx laminae was assumed to be transversely isotropic:

(11) $$E_{2}=E_{3}=E_{m}\left(\frac{1+\zeta \eta V_{f}}{1-\eta V_{f}}\right)$$

where

(12) $$\eta =\frac{\left(E_{f}/E_{m}\right)-1}{\left(E_{f}/E_{m}\right)+\zeta }\;\;\;;\;\;\;\zeta =2+40V_{r}^{10}$$

*G_n_^C^*, *G_s_^C^,* and *G_t_^C^* were defined by the experimental study of Santos et al. [16] on the interlaminar fracture toughness of a 3D-printed continuous carbon fibre-reinforced composite. The assumption for *T_s_*^0^ = *T_t_*^0^ was made, and *T_s_*^0^, which had been obtained via three-point short beam tests of a stacking sequence made of UD carbon/nylon laminae by the effort of Yavas et al. [15], was selected. *T_n_*^0^ was generated by an equation of *T_s_*^0^, *G_n_^C^*, and *G_s_^C^* [33]:

(13) $$T_{n}^{0}={\left(\frac{G_{n}^{C}}{G_{s}^{C}}\right)}^{\frac{1}{2}}T_{s}^{0}$$

*G_n_^C^*, *G_s_^C^* = *G_t_^C^*, and *T_s_*^0^, defined above, had been used in Ref. [2], which focused on an interaction between continuous UD carbon/nylon lamina and Nylon White. The calculated *k_nn_*, *k_ss_*, and *k_tt_* of this study decreased by 20% for the L-T1-C and H-T1-C batches and 4% for the H-T2-C batch, respectively, when they were compared with the *k_nn_*, *k_ss_*, and *k_tt_* of Ref. [2]. As a result, *G_n_^C^*, *G_s_^C^* = *G_t_^C^*, and *T_s_*^0^ were modified coarsely by having the same decrease rates in order to have a better prediction of the finite element method (FEM) in this study. Table 6 summarises the predicted interfacial properties of the cohesive contact between a UD carbon/nylon and an Onyx lamina for all selected batches. The viscosity coefficient is used to regularise the convergence, and it was set to 0.0001 in Abaqus.

**Figure 9:**
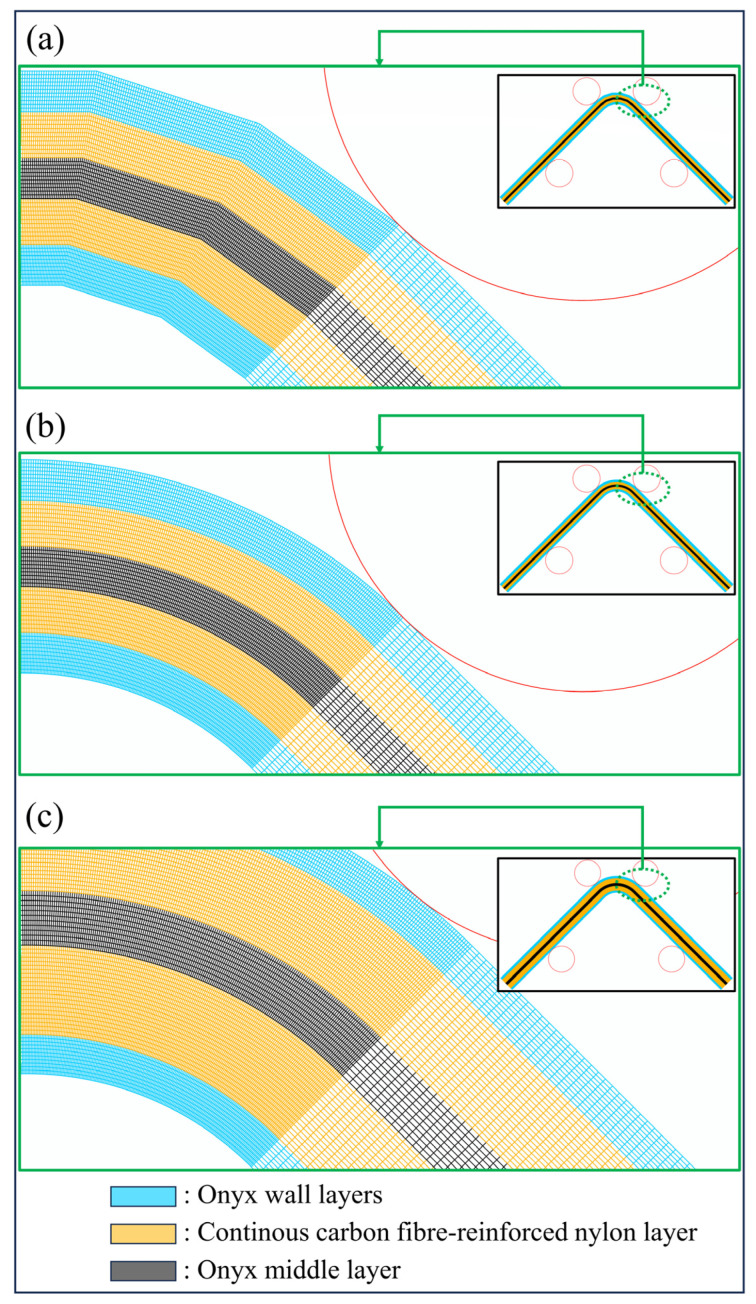
The 2D finite element model and the detailed curved region mesh of (**a**) the L-T1, (**b**) the H-T1, and (**c**) the H-T2 batch.

The 2D models of the beams and rollers were meshed using four-node plane strain elements with reduced integration (CPE4R) and two-node 2D linear rigid elements (R2D2), respectively. Sensitivity analysis was performed for a similar model by making a wide range of cases run, and convergence was achieved for the following case [2]. The approximate global mesh size was assigned as 0.2 mm for both beam cross-sections and rollers. A total of 55 and 77 elements were additionally defined through the thickness of T1 and T2, respectively, by sharing the total number equally for each lamina. Local intensive mesh size for the critical curved region, which was assigned as the total element number of 360 along the curved edges of each lamina. The finite element models, which depend on mesh parameters, are shown in Figure 9. The mesh sizes in the models for L-T1-C, H-T1-C, and H-T2-C batches contained 53,952, 54,816, and 73,736 elements, respectively.

## 3. Results and Discussion

### 3.1. Print Resolution Effect

**Figure 10:**
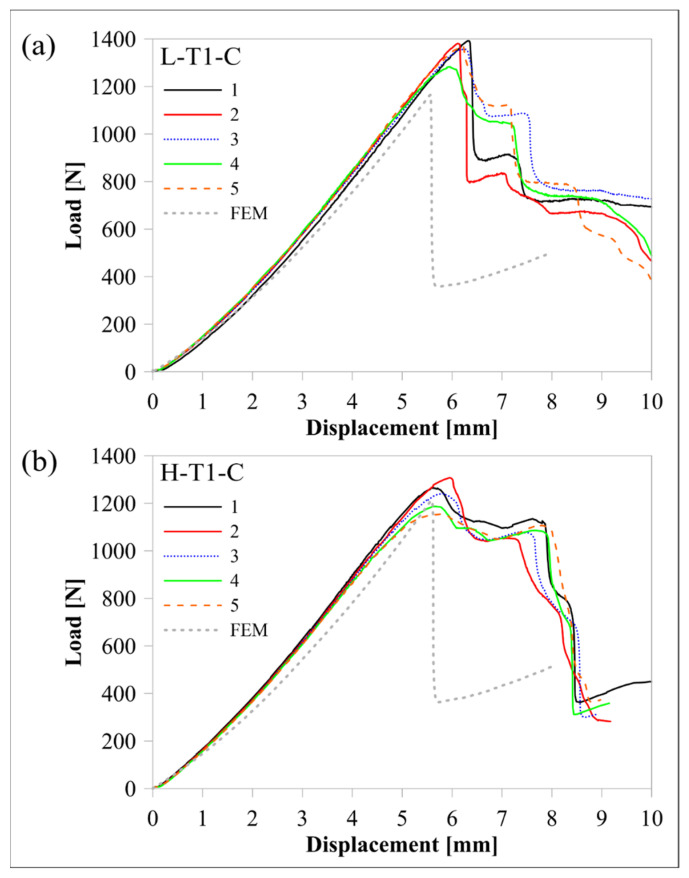
Comparison of experimental and numerical load-displacement histories between (**a**) the L-T1-C batch and (**b**) the H-T1-C batch.

**Figure 11:**
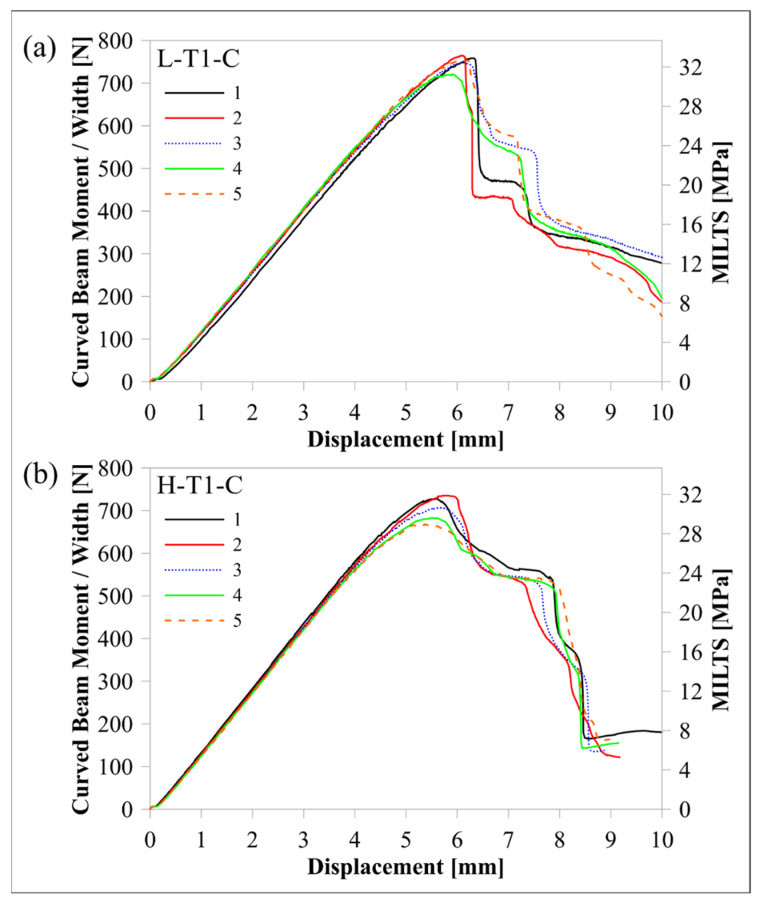
Comparison of M/w-displacement and MILTS-displacement histories between (**a**) the L-T1-C batch and (**b**) the H-T1-C batch.

The response of the L-T1-C design, whose curved region had five flat zones for all laminae, was compared with the response of the H-T1-C design, which had a smooth curved region. Figure 10 and Figure 11 compare load-displacement and M/w-displacement with MILTS-displacement histories of L-T1-C specimens with the histories of H-T1-C specimens, respectively. The main trend shows that most of the L-T1-C specimens had a higher maximum load, CBS, and ILTS than the H-T1-C specimens. L-T1-C specimens’ displacements at maximum load were slightly higher than H-T1-C specimens’ displacements. The reason for having higher values was quite likely having better cohesive properties between the continuous carbon/nylon and Onyx laminae in L-T1 samples when the interfaces were flat. A similar result had been obtained in another study by Arki et al. [34], who investigated a conventionally manufactured UD carbon fibre-reinforced composite curved beam subjected to bending loading. The M-shaped curved beam, which had a main flat region and two curved parts linked by that flat area, achieved a 15% increase in CBS by comparing it with the smooth curved region. It was explored that additively manufactured FPL designs in this study had similar results to moulding-based composite laminated composite designs. However, a negative correlation between the number of flat zones in the curved region and the strength can be presented in the literature for discussion in future studies.

On the other hand, the H-T1-C design was superior to the L-T1-C design in terms of durability for preserving load-carrying capacity. The load and strength curves of L-T1 batch specimens had a sudden big drop after the peak, while H-T1 specimens resist high stress better and retard failure at a higher displacement. Stress concentration, which led to higher local stress on the L-T1 batch, made the specimens less durable.

Predicted load-displacement curves of finite element models are also shown in Figure 10. Load was calculated by multiplying the sum of the reaction force in the upper rollers in a 2D model by 23.75 mm, which is the *w* of the beam without Onyx floor and roof layers. There is better agreement between the experimental and numerical results of the H-T1 model than the L-T1 model. It should be kept in mind that only the geometric effect of flat portions in the curved region was included in the model, while the same surface interaction properties were used for both models. The newly modified cohesive properties for the L-T1 model will lead to better agreement for maximum load and displacement at maximum load. Although the H-T1 model was good at achieving agreement for peak load and displacement, the trend line after the peak load suggested a sudden drop, unlike experimental results. The post-delamination trend line of the L-T1 model was agreeable, apart from predicting the start of delamination earlier than experimental results.

Table 7 compares the mean values of maximum load, CBS, and ILTS for each batch and compares them with finite element results. The difference in strength capacity between L-T1 and H-T1 batch results is clearer in Table 7. Five flat zones in the curved region achieved 10% and 6% increases in maximum load and ILTS, respectively, when compared with a smooth curved region. The percentage differences of finite element results with experimental ones for maximum load and ILTS were found to be 14% and 3%, respectively, for the L-T1 finite element model. The percentage difference for the ILTS value was superior to the one for the maximum load in the same numerical model. Moreover, percentage differences were perfectly small (2% for the maximum load and 1% for ILTS) for the H-T1 numerical model. It means the generated interaction properties between UD carbon/nylon and Onyx laminae in Table 6 worked better on a smooth, curved region.

**Figure 12:**
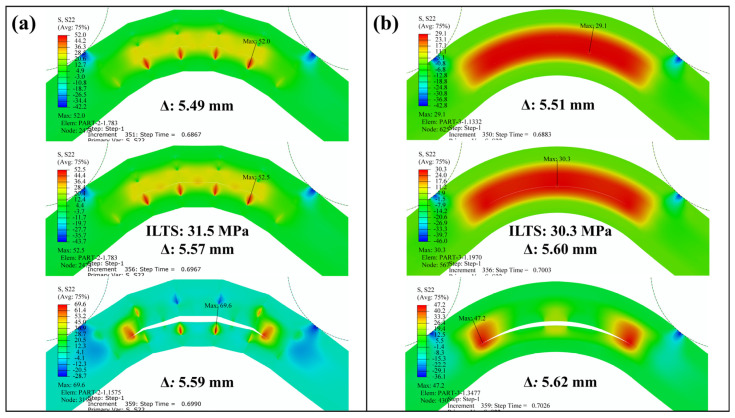
Interlaminar tensile stress distribution in the curved region and the delamination propagation in (**a**) the L-T1-C and (**b**) the H-T1-C finite element models.

Interlaminar tensile stress distributions for both finite element models were obtained and are monitored at three certain Δ, including the start of delamination in Figure 12, via contour plots. There was a distinctive change between the stress distributions of the curved regions, which approved the stress concentrations in the L-T1 batch results above. ILTS of the L-T1 numerical model was defined as 31.5 MPa in Table 7, which was the maximum stress between the lower carbon/nylon and middle Onyx laminae. Figure 12a shows the local tensile stress reached up to 52.5 MPa. On the other hand, the H-T1 model represented a balanced stress distribution dominantly carried by both carbon/nylon laminae and middle Onyx laminae. The radial damage location (*r_d_*) was approved as 8.1 mm since the delamination occurred in the surface interaction between the lower carbon/nylon lamina and middle Onyx lamina for both numerical models.

**Figure 13:**
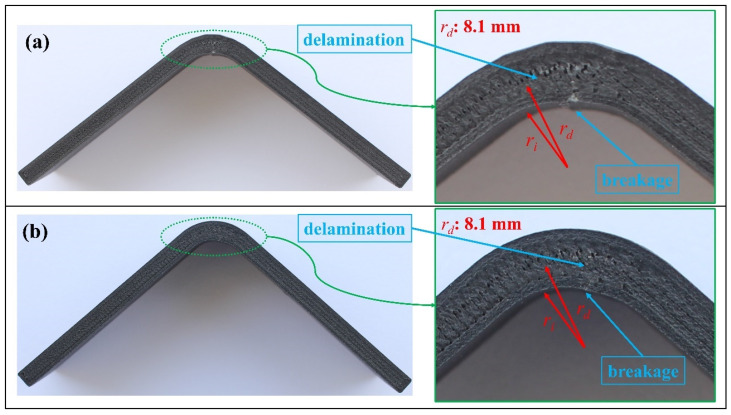
Damage analysis on the image of (**a**) the L-T1-C and (**b**) the H-T1-C test specimen.

Damage mechanisms for both curved beams are shown in Figure 13. The *r_d_* values are consistent with finite element results. Tests were mainly carried on until Δ reached 10 mm to observe the post-delamination behaviours. The breakage in lower Onyx wall layers occurred due to excessive tensile strain after delamination, while FEM did not simulate this breakage.

### 3.2. Continuous Fibre Material Effect

**Figure 14:**
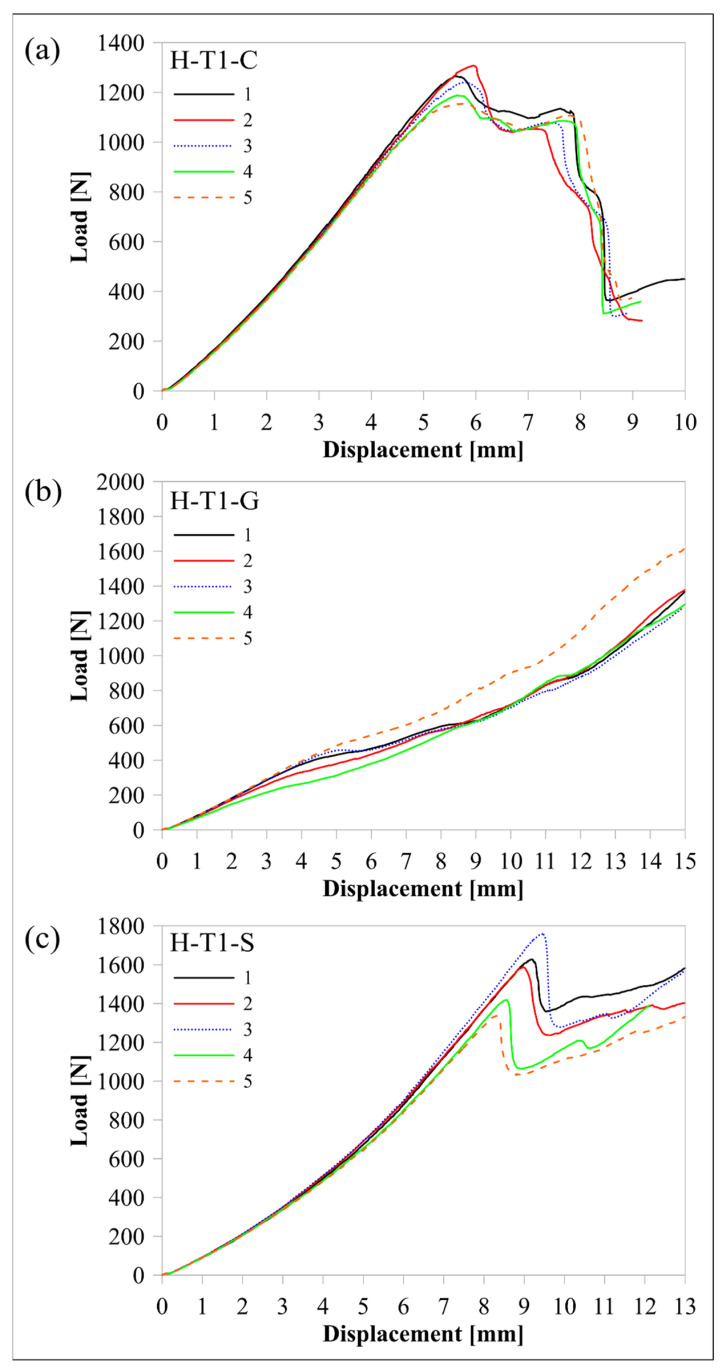
Comparison of load-displacement histories among (**a**) the H-T1-C, (**b**) the H-T1-G, and (**c**) the H-T1-S batch.

**Figure 15:**
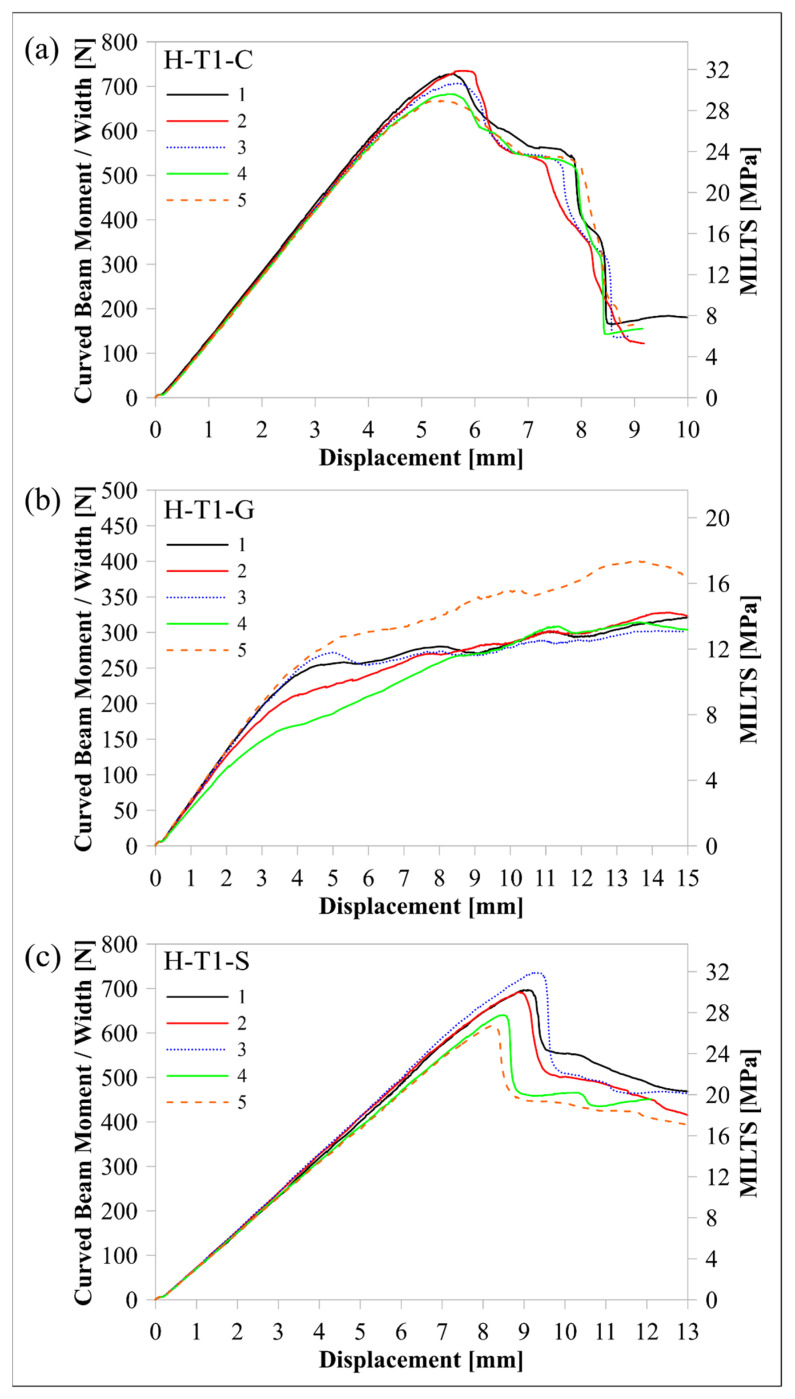
Comparison of M/w-displacement and MILTS-displacement histories among (**a**) the H-T1-C, (**b**) the H-T1-G, and (**c**) the H-T1-S batch.

After the effect of flat zones in the curved region had been analysed, H-T1 beam designs made of certain continuous fibre-reinforced nylon and Onyx laminae were compared. Figure 14 and Figure 15 compare load-displacement and M/w-displacement with MILTS-displacement histories among the C, G, and S batches of H-T1 specimens, respectively. The experimental results are also summarised in Table 8 to define the mean values of their maximum loads, CBS and ILTS. Specific ILTS (ILTS to part mass ratio) and ILTS to material cost ratio are also included in Table 8.

Specimens of the H-T1-S batch succeeded in a load drop to calculate CBS and ILTS values, while the H-T1-G batch had no drop in force or multiple force drops, although the tests continued until Δ reached 15 mm (*φ* is less than 5° at Δ: 15 mm). The mean values of CBS and ILTS for the H-T1-G batch are therefore shown as less than a certain value in Table 8 to compare them with the values of the other two batches. CBS and ILTS of the H-T1-S batch look quite competitive with the ones for the H-T1-C batch, unlike H-T1-G, as shown in Table 8. H-T1-S batch even has better performance when cost is an important concern according to its ILTS to material cost ratio. Changing the continuous UD fibre material from carbon to highly strengthened glass fibre led to a decrease of only around 4% in strength, while the decrease became higher (10%) in specific strength since glass has a higher density than carbon. Using highly strengthened glass fibres instead of conventional glass fibres doubled the ILTS value.

**Figure 16:**
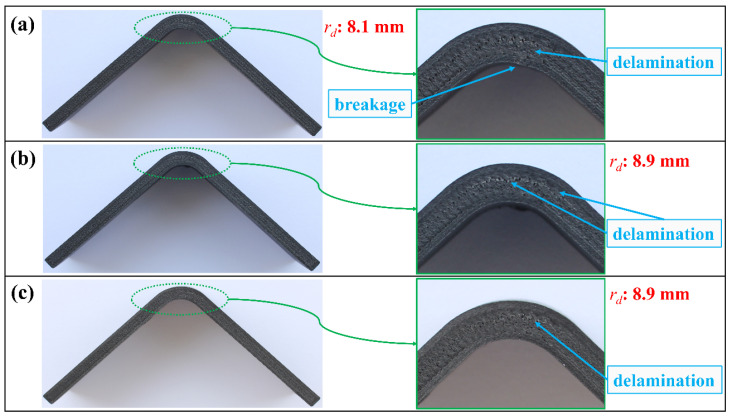
Damage analysis on the image of (**a**) the H-T1-C, (**b**) the H-T1-G, and (**c**) the H-T1-S test specimen.

When the damage characteristics of all batches are examined in Figure 16, both H-T1-G and H-T1-S batches obviously had the presence of delamination like the H-T1-C batch. However, the *r_d_* values of these batches are 8.9 mm, and the location is between the upper continuous UD lamina and middle Onyx lamina, unlike the *r_d_* of H-T1-C. Moreover, there is no breakage in the lower Onyx wall layers of the G and S reinforced batches. H-T1-G noticeably had delamination in Figure 16b, but the test results of this batch in Figure 14b showed no sign of the damage mechanism.

### 3.3. Thickness Effect

**Figure 17:**
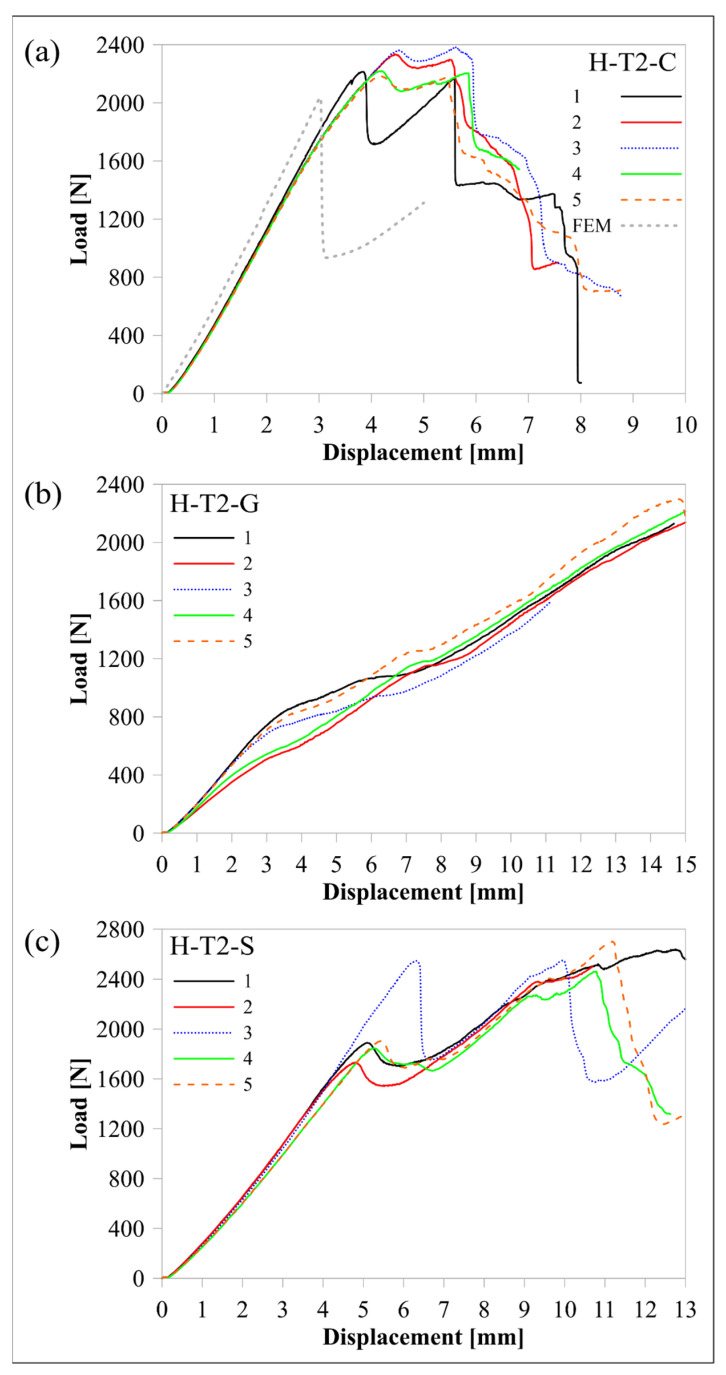
Comparison of load-displacement histories among (**a**) H-T2-C, (**b**) H-T2-G, and (**c**) H-T2-S batches.

**Figure 18:**
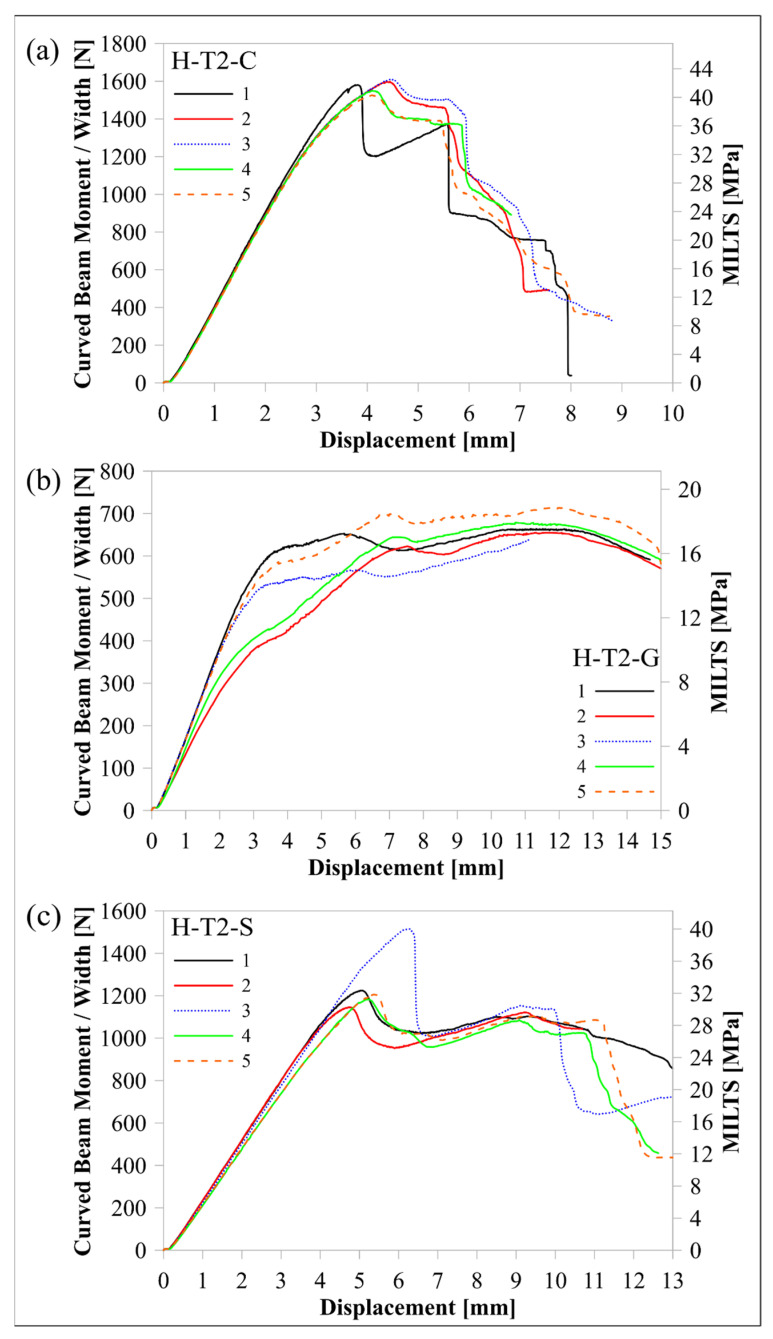
Comparison of M/w-displacement and MILTS-displacement histories among (**a**) H-T2-C, (**b**) H-T2-G, and (**c**) H-T2-S batches.

The continuous fibre material effect was modified, and the thickness effect was included in the previous case by increasing the thickness of the upper and lower carbon/nylon laminae by 100% and the middle Onyx lamina by 22% (a total increase in the beam thickness of 50%). Figure 17 shows load-displacement histories of the H-T2-C, H-T2-G, and H-T2-S specimens, while Figure 18 shows M/w-displacement and MILTS-displacement histories of the same batches’ specimens.

The main trend of the load-displacement curves changed for H-T2’s histories except for the H-T2-G batch when the histories were compared with H-T1’s histories. Load-displacement histories had double peaks. The first peak is higher for the H-T2-C batch, and vice versa for the H-T2-S batch. Increasing the thickness of the curved beam by doubling glass fibre-reinforced nylon laminas resulted in a 50% increase in *V_r_*. However, the contribution to stiffness did not affect observing the force drop of each H-T2-G specimen in load-displacement histories. The average displacement at maximum load for H-T2 batches was smaller than the H-T1 design, as expected since the specimens became stiffer.

The load-displacement curve of the H-T2-C finite element model is also shown in Figure 17a to compare it with experimental results and the T1-C finite element models in Figure 10. The rate of agreement with experimental results looked similar to the L-T1-C model. The post-delamination trend had an agreement, apart from predicting an earlier start of delamination than experimental results.

The mean values of the maximum load, CBS, ILTS, specific ILTS, and cost effectiveness for the tests of all H-T2 batches and the finite element results of the H-T2-C model are compared in Table 9. Changing the continuous UD fibre material from carbon to high-strength, high-temperature glass fibre caused a decrease of around 20% in ILTS, while the decrease rate was higher for specific ILTS. Increasing the thickness of high-strengthened glass fibre-reinforced nylon made the S-reinforced design less challenging than the C-reinforced design since H-T1-S had a decrease of 4% against H-T1-C. The thickness effect led to an increase of 36% and 13% in strength for the C-reinforced design and for the S-reinforced design, respectively. On the contrary, specific strength values decreased by 10% and 26% for H-T2-C and H-T2-S, respectively. Moreover, decreases in ILTS to cost ratios of T2 batches looked much worse than specific ILTS. The H-T2-G design had no sign of being competitive with other batches like the H-T1-G design.

**Figure 19:**
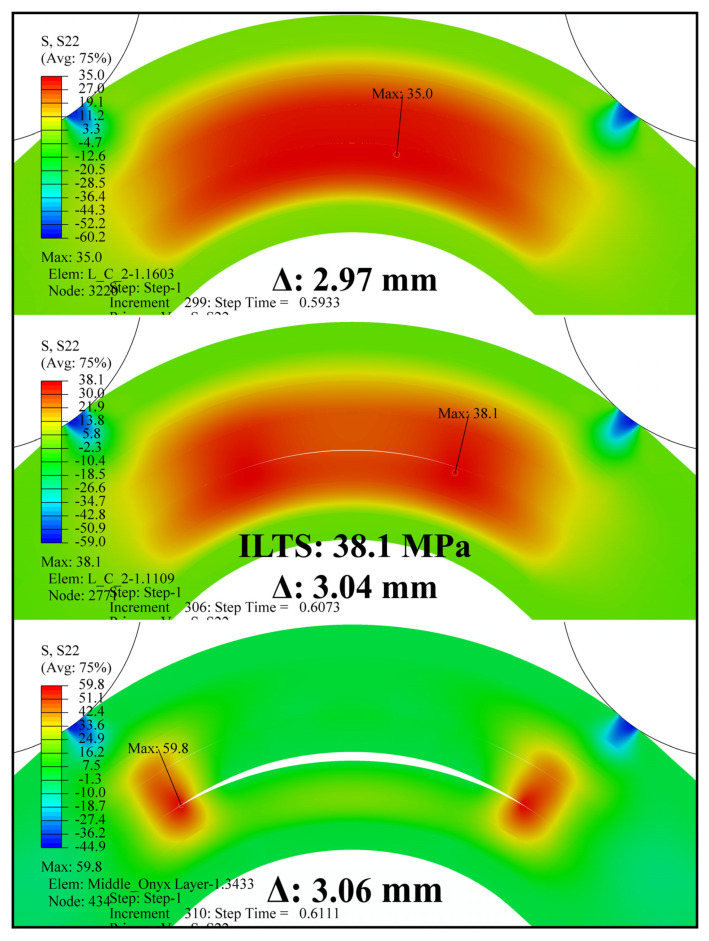
Interlaminar tensile stress distribution in the curved region and delamination propagation in the H-T2-C finite element model.

The maximum load and ILTS values obtained by the H-T2-C finite element model are also shown in Table 9. The percentage differences between finite element results and experimental ones for maximum load and ILTS are found to be 10% and 8%, respectively. Interlaminar tensile stress distribution in the curved region and the delamination propagation at *r_d_*: 9.0 mm (between the second lower carbon/nylon lamina and middle Onyx lamina) are shown at three certain Δ values (on the edge of delamination, delamination, and after the start of delamination) in Figure 19. The H-T2-C model simulated a balanced stress distribution dominantly carried by both carbon/nylon laminae and middle Onyx laminae, similar to the H-T1-C design.

**Figure 20:**
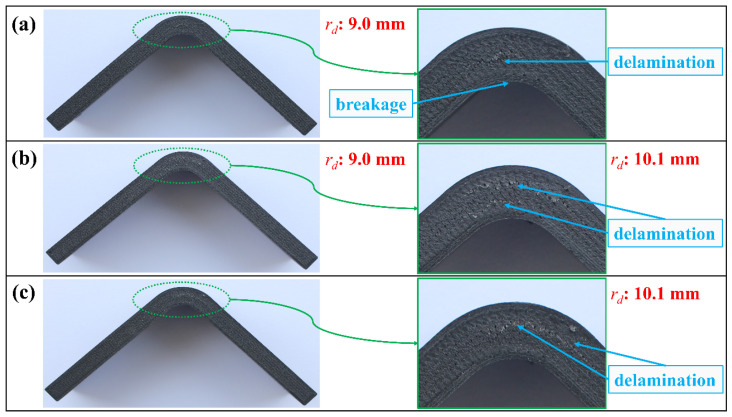
Damage analysis on the image of (**a**) H-T2-C, (**b**) H-T2-G, and (**c**) H-T2-S test specimen.

The damage characteristics on the image of the H-T2-C test specimen in Figure 20a and the H-T1-C test specimen in Figure 16a are the same. Although the *r_d_* values are different due to changes in the thickness, the delamination occurred between the middle Onyx lamina and the adjacent lower carbon/nylon lamina for both batches. A similar inference can be drawn for the damage analysis of H-T2-S after comparing it with H-T1-S in Figure 16c and Figure 20c. The damage characteristic of the H-T2-G specimen in Figure 20b shows a different pattern against all other H-T1 and H-T2 batches. A double delamination characteristic at both *r_d_*: 9.0 mm and *r_d_*: 10.1 mm occurred. However, this damage mechanism unexpectedly hid in the load-displacement curves in Figure 17b as the H-T1-G batch.

### 3.4. Onyx versus Nylon White

**Figure 21:**
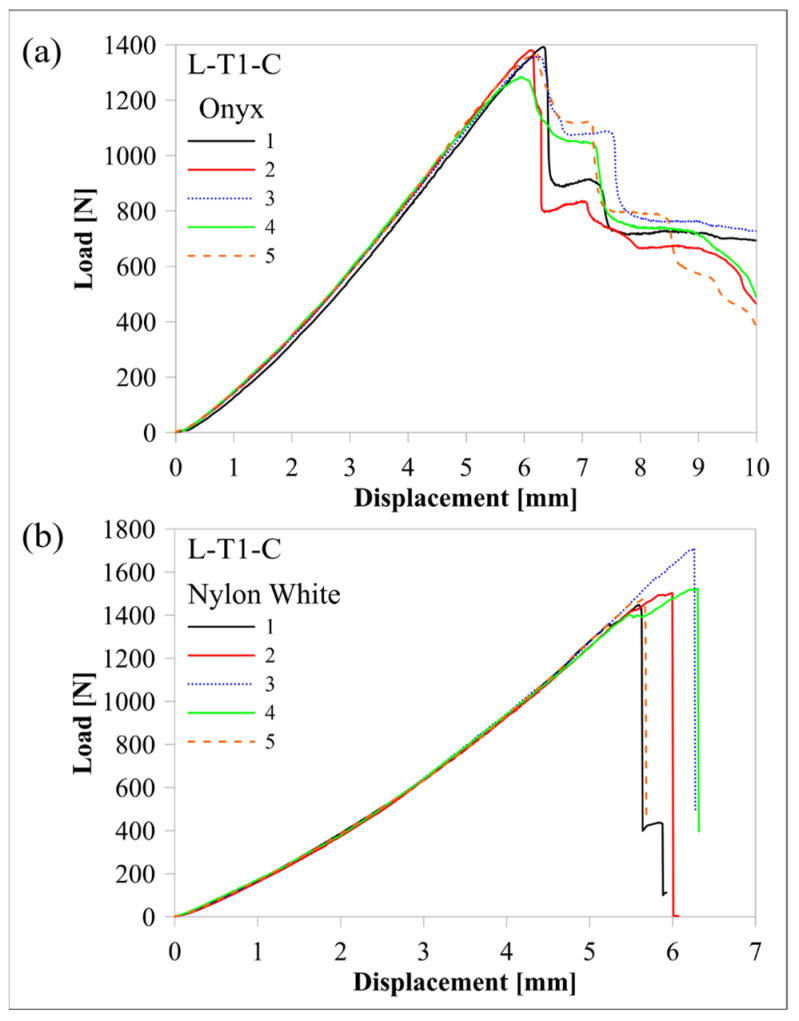
Comparison of load-displacement histories between (**a**) the batch of L-T1-C with Onyx polymer laminae and (**b**) [2] the batch of L-T1-C with Nylon White laminae.

**Figure 22:**
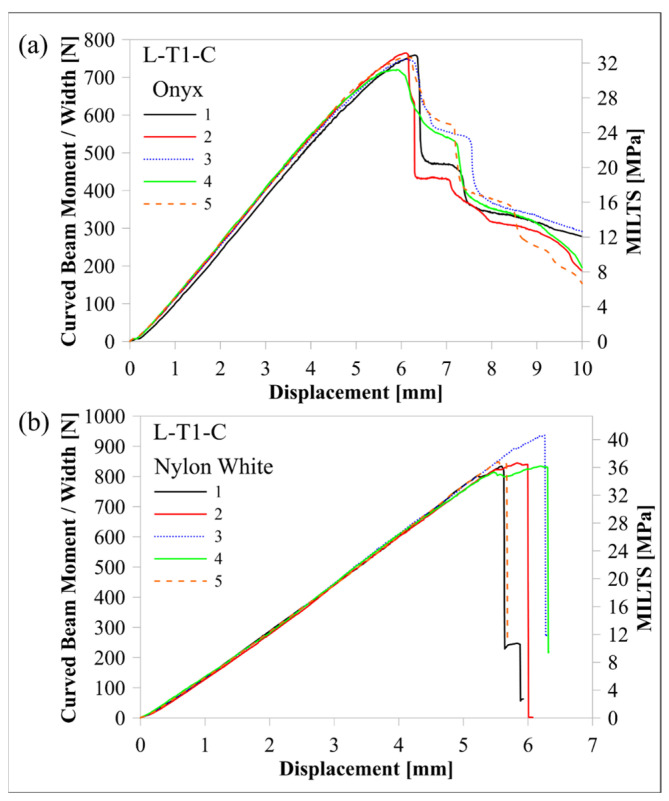
Comparison of M/w-displacement and MILTS-displacement histories between (**a**) the batch of L-T1-C with Onyx polymer laminae and (**b**) [2] the batch of L-T1-C with Nylon White laminae.

It is meaningful to explore the comparison of curved beam strength if Nylon White laminae are replaced with Onyx laminae in an FPL design. It is also a comparison between a hybrid design and a non-hybrid design. Nylon White is stronger and stiffer than conventional nylon for 3D printing without short carbon fibre reinforcement, unlike Onyx. Figure 21 and Figure 22 compare the load, CBS, and strength curves of L-T1-C specimens in this study with the curves of L-T1-C specimens in Ref. [2]. The comparative maximum load, CBS, and ILTS mean values of test results for the discussion on the effect of using Onyx instead of Nylon White for the material of the middle Onyx lamina and Onyx wall layers are also shown in Table 10.

When a polymer material is reinforced with short carbon fibres, it is supposed to contribute more strength to a structure. However, the L-T1-C–Onyx design had a decrease in the maximum load and the ILTS by 11% and 13%, respectively. The reason was most probably that the existence of short carbon fibres in Onyx decreased the adhesion strength. A stiffer and more adhesive nylon was superior to a short fibre-reinforced, less adhesive nylon in the characteristic of delamination damage. On the other hand, another challenging ability needs to be analysed in terms of durability. Although the L-T1-C–Onyx design had lower strength properties, load carrying capacity and strength were preserved superiorly to L-T1-C–Nylon White. There was a fair amount of drop and then a plateau in load against displacement for Onyx-based specimens, while the Nylon White-based specimens had an excessively sharp load drop to zero. Moreover, the displacement of both designs at maximum load was so close to each other.

## 4. Conclusions

In this paper, the delamination strength capacities of additively manufactured hybrid composite curved beams, which were in the form of FPL designs, were explored by creating various concepts of comparison. Print resolution, which created flat zones in the curved region when low print resolution was selected, had a significant effect on not only load carrying capacity but also CBS and ILTS. Generally, the 3D printing industry recommends manufacturing the curved parts in high resolution (suggested angular tolerance of 1°) [35]. Our study showed that low-resolution manufacturing can be advantageous to obtain better results in the case of delamination strength if 3D-printed brackets or fitting parts need flat transition regions for installation. However, it is important to note that stress concentration becomes a significant concern when dealing with flat portions. This is particularly crucial due to the durability issues that may arise, affecting the load or stress-carrying capacity post-delamination initiation.

Exploring the continuous UD fibre material effect on the performance of an FPL hybrid curved beam showed that embedding continuous carbon/nylon laminae is inevitably more effective than the other glass fibre-based 3D-printing filaments. However, high-strength, high-temperature glass filament is obviously competitive with the performance of carbon/nylon filament on CBS and ILTS. Stiffness, flexibility, and the higher cost of carbon filament will be other parameters to consider. The selection of continuous fibre material became indicative of the damage characteristic and even of the radial location of delamination. Moreover, having a higher maximum load is not a criterion for having higher CBS and ILTS in this test method since the displacement at the maximum load is really important. Additionally, the test method can be performed inefficiently on structures that do not have enough stiffness. The batches, which are H-T1-G and H-T2-G, had acceptable damage, which was hidden in load-displacement histories.

At first glance, increasing the thickness of the whole curved beam by mainly printing double continuous fibre-reinforced laminae created a great increase in the strength of all FPL designs, especially carbon/nylon laminae-based designs. When the part mass and material cost were taken into account, increasing the number of UD continuous fibres had no effect on having better performance. However, using strengthened glass/nylon FPL in design lost its previous competitive performance against using carbon/nylon.

Finite element models were created and analysed to search for the availability of useful virtual testing with consistent material and cohesive properties. The lowest percentage differences were obtained for the H-T1-C model, although there is generally good agreement with experimental results for all finite element models. FEM predicted damage locations consistent with experimental results except for the Onyx breakage in lower wall layers. FEM analysed interlaminar stress distribution, unlike an analytically based experimental study, and contributed to comparing the responses of flat-zoned and smooth-curved regions, especially. In this study, generating surface interaction properties is built on a simple idea that creates a correlation between each model. Small modifications could have worked great for the finite element models, which had less agreement with experimental results in future studies.

L-T1 batches made of various continuous fibre-reinforced nylon and Nylon White laminae had been deeply investigated before by the authors. Onyx versus Nylon White was a good challenge under these circumstances to link these studies. Having Nylon White laminae in an FPL design instead of Onyx material made the curved beam stronger. On the other hand, the curved beam with Onyx laminae contributed to preserving a significant amount of load at an acceptable displacement value, while the existence of Nylon White laminae was prone to catastrophic failure for a structure.

This study will greatly contribute to future studies that will focus on 3D-printed composite curved beams, both numerically and experimentally. Projects on designing and 3D printing structural composite parts like brackets and fittings will uncover valuable time-saving data. For future work, it would be interesting to investigate the effect of including non-unidirectional continuous fibre-reinforced laminae in 3D-printed composite curved beams experimentally and numerically. Modelling additively manufactured FPL curved parts by the finite difference method and the Bézier-based multi-step method and comparing them with FEA would be a powerful numerical study.

## Figures and Tables

**Table 1:** The printing inputs.

Parameter	Input
Fill Pattern	Solid Fill
Fill Density	100%
Wall Layers	2
Fibre Pattern Type	Entire Group
Fibre Fill Type	Concentric Fibre
Concentric Fibre Rings	1 (for T1)
	2 (for T2)
Start Rotation Percent	0
Use Supports	Raise Part
Use Brim	Yes

**Table 2:** Understanding the codes of the specimens.

Sub-Code	Description
L	Low Resolution (Five-Flat Zoned Curved Region)
H	High Resolution (Smooth Curved Region)
T1	4.2 mm Thickness-Curved Beam
T2	6.3 mm Thickness-Curved Beam
C	Continuous UD Carbon Fibre-Reinforced Nylon
G	Continuous UD Glass Fibre-Reinforced Nylon
S	Continuous UD High Strength High Temperature Glass Fibre-Reinforced Nylon

**Table 3:** The printing outputs.

Batch	Part Mass	Material Cost	*υ_m_*	*υ_f_*	*V_r_*
	[g]	[$]	[cm^3^]	[cm^3^]	[%]
L-T1-C	15.77	17.37	12.10	4.83	28.5
H-T1-C	15.79	17.38	12.11	4.83	28.5
H-T1-G	16.85	10.69	12.14	4.88	28.7
H-T1-S	16.85	12.65	12.14	4.88	28.7
H-T2-C	23.69	32.32	12.98	9.75	42.9
H-T2-G	25.72	18.80	12.94	9.83	43.2
H-T2-S	25.72	22.74	12.94	9.83	43.2

**Table 4:** The test matrix.

Batch ID	No. of Flat Zones	UD Fibre Material	*t*	No. of Specimens	Placement (*l_b_*-*l_t_*) [mm]	Testing Speed [mm/min]	Sampling Rate [Hz]
1	5	Carbon	T1	5	42.1–22.1	3	10
2	90	Carbon	T1				
3	90	Glass	T1				
4	90	S-Glass	T1				
5	90	Carbon	T2				
6	90	Glass	T2				
7	90	S-Glass	T2				

**Table 5:** Material properties of the constituents.

Material	*E*_1_	*E*_2_	*ν*_12_	*G*_12_	UTS
	[GPa]	[GPa]		[GPa]	[MPa]
UD Carbon/Nylon [29]	35.00	7.50	0.15	4.00	-
Onyx [21]	1.18	1.18	0.41	-	39.98

**Table 6:** Surface interaction properties for certain batches.

		L-T1-C and H-T1-C	H-T2-C
*k_nn_ = k_ss_* = *k_tt_*	[MPa/mm]	116,000	140,000
*G_n_^C^*	[mJ/mm^2^]	1.4	1.6
*G_s_^C^* = *G_t_^C^*	[mJ/mm^2^]	1.8	2.2
*m*		2	2
*T_n_* ^0^	[MPa]	28.8	33.8
*T_s_*^0^ *= T_t_*^0^	[MPa]	32.7	39.3

**Table 7:** The comparative maximum load, CBS and ILTS mean values of test results, and FEM for the discussion on the effect of flat zones in the curved region.

Batch	Method	Maximum Load [N]	CBS [N]	ILTS [MPa]
L-T1-C	Test	1357	749	32.5
	FEM	1165	n/c	31.5
H-T1-C	Test	1231	704	30.6
	FEM	1212	n/c	30.3

**Table 8:** The comparative maximum load, CBS, ILTS, specific ILTS, and ILTS to cost ratio mean values of test results for the discussion on the effect of continuous UD fibre material used in the curved beam.

Batch	Method	Maximum Load [N]	CBS [N]	ILTS [MPa]	Specific ILTS [MPa/g]	ILTS/Cost [MPa/$]
H-T1-C	Test	1231	704	30.6	1.94	1.76
H-T1-G	Test	n/c	<334	<14.4	<0.85	<1.35
H-T1-S	Test	1546	676	29.3	1.74	2.32

**Table 9:** The comparative maximum load, CBS, ILTS, specific ILTS, and ILTS to cost ratio mean values of test results and FEM for the discussion on the effect of increasing the thickness of continuous fibre-reinforced nylon.

Batch	Method	Maximum Load [N]	CBS [N]	ILTS [MPa]	Specific ILTS [MPa/g]	ILTS/Cost [MPa/$]
H-T2-C	Test	2267	1573	41.5	1.75	1.28
	FEM	2037	n/c	38.1	n/c	n/c
H-T2-G	Test	n/c	<670	<17.7	<0.69	<0.94
H-T2-S	Test	2572	1255	33.1	1.29	1.46

**Table 10:** The comparative maximum load, CBS, and ILTS mean values of test results for the discussion on the effect of the polymer laminae material.

Batch	Method	Maximum Load [N]	CBS [N]	ILTS [MPa]
L-T1-C–Onyx	Test	1357	749	32.5
L-T1-C–Nylon White [2]	Test	1532	860	37.3

## Data Availability

The data presented in this study are available on request from the corresponding author. The data are not publicly available due to privacy.

## References

[1] Brenken B., Barocio E., Favaloro A., Kunc V. (2018). Fused filament fabrication of fiber-reinforced polymers: A review. Addit. Manuf..

[2] Süsler S., Kazancı Z. (2023). Delamination strength comparison of additively manufactured composite curved beams using continuous fibers. Polymers.

[3] Stamopoulos A.G., Glinz J., Senck S. (2024). Assessment of the effects of the addition of continuous fiber filaments in PA 6/short fiber 3D-printed components using interrupted in-situ X-ray CT tensile testing. Eng. Fail. Anal..

[4] Fijul Kabir S.M., Mathur K., Seyam A.F.M. (2020). A critical review on 3D printed continuous fiber-reinforced composites: Histo-ry, mechanism, materials and properties. Compos. Struct..

[5] Handwerker M., Wellnitz J., Marzbani H. (2021). Review of mechanical properties of and optimisation methods for continuous fibre-reinforced thermoplastic parts manufactured by fused deposition modelling. Prog. Addit. Manuf..

[6] Jamal M.A., Shah O.R., Ghafoor U., Qureshi Y., Bhutta M.R. (2024). Additive manufacturing of continuous fiber-reinforced polymer composites via fused deposition modelling: A comprehensive review. Polymers.

[7] Shiratori H., Todoroki A., Ueda M., Matsuzaki R., Hirano Y. (2020). Mechanism of folding a fiber bundle in the curved section of 3D printed carbon fiber reinforced plastics. Adv. Compos. Mater..

[8] Shiratori H., Todoroki A., Ueda M., Matsuzaki R., Hirano Y. (2021). Compressive strength degradation of the curved sections of 3D-printed continuous carbon fiber composite. Compos. Part A Appl. Sci. Manuf..

[9] Shiratori H., Todoroki A., Ueda M., Matsuzaki R., Hirano Y. (2021). Testing method for evaluating mechanical properties of 3D printed CFRP with curved fibers by four-point bending test of L-shaped specimen. Compos. Part C Open Access.

[10] Standard Test Method for Short-Beam Strength of Polymer Matrix Composite Materials and Their Laminates.

[11] Standard Test Method for Measuring the Curved Beam Strength of a Fiber-Reinforced Polymer-Matrix Composite.

[12] Yang C., Tian X., Liu T., Cao Y., Li D. (2017). 3D printing for continuous fiber reinforced thermoplastic composites: Mechanism and performance. Rapid Prototyp. J..

[13] Caminero M.A., Chacón J.M., García-Moreno I., Reverte J.M. (2018). Interlaminar bonding performance of 3D printed continuous fibre reinforced thermoplastic composites using fused deposition modelling. Polym. Test..

[14] Iragi M., Pascual-González C., Esnaola A., Lopes C.S., Aretxabaleta L. (2019). Ply and interlaminar behaviours of 3D printed continuous carbon fibre-reinforced thermoplastic laminates; effects of processing conditions and microstructure. Addit. Manuf..

[15] Yavas D., Zhang Z., Liu Q., Wu D. (2021). Interlaminar shear behavior of continuous and short carbon fiber reinforced polymer composites fabricated by additive manufacturing. Compos. Part B Eng..

[16] Santos J.D., Fernández A., Ripoll L., Blanco N. (2022). Experimental characterization and analysis of the in-plane elastic properties and interlaminar fracture toughness of a 3D-printed continuous carbon fiber-reinforced composite. Polymers.

[17] Damodaran V., Castellanos A.G., Milostan M., Prabhakar P. (2018). Improving the mode-II interlaminar fracture toughness of polymeric matrix composites through additive manufacturing. Mater. Des..

[18] Touchard F., Arnault L.C., Fournier T., Magro C., Lafitte A., Caradec A. (2021). Interfacial adhesion quality in 3D printed continuous CF/PA6 composites at filament/matrix and interlaminar scales. Compos. Part B Eng..

[19] Gonzalez C.P., San Martin P., Lizarralde I., Fernandez A., Leon A., Lopes C.S., Fernandez-Blazques J.P. (2021). Post-processing effects on microstruture, interlaminar and thermal properties of 3D printed continous carbon fibre composites. Compos. Part B Eng..

[20] Santos J.D., Guerrero J.M., Blanco N., Fajardo J.I., Paltan C.A. (2023). Numerical and experimental analysis of the mode I interlaminar fracture toughness in multidirectional 3D-printed thermoplastic composites reinforced with continuous carbon fiber. Polymers.

[21] Fisher T., Almedia J.H.S., Falzon B.G., Kazancı Z. (2023). Tension and compression properties of 3D-printed composites: Print orientation and strain rate effects. Polymers.

[22] Dassault Systemes Solidworks 2021 Software. https://www.solidworks.com/.

[23] Markforged Eiger Software. https://www.eiger.io/.

[24] Markforged Mark Two 3D Printer. https://markforged.com/3d-printers/mark-two.

[25] Lloyd Instruments Lloyd LS5 Testing Machine. https://www.ametektest.com/products/material-testers/single-column-test-stands/ls-series.

[26] Wang Y., Gu Y., Liu J. (2020). A domain-decomposition generalized finite difference method for stress analysis in three-dimensional composite materials. Appl. Math. Lett..

[27] Kabir H., Aghdam M.M. (2021). A generalized 2D Bézier-based solution for stress analysis of notched epoxy resin plates reinforced with graphene nanoplatelets. Thin-Walled Struct..

[28] Simulia Abaqus/CAE 2021 Software. https://www.3ds.com/products-services/simulia/products/abaqus/.

[29] Galati M., Viccica M., Minetola P. (2021). A finite element approach for the prediction of the mechanical behaviour of layered composite produced by continuous filament fabrication (CFF). Polym. Test..

[30] Benzeggagh M.L., Kenane M. (1996). Measurement of mixed-mode delamination fracture toughness of unidirectional glass/epoxy composites with mixed-mode bending apparatus. Compos. Sci. Technol..

[31] Turon A., Davila C.G., Camanho P.P., Costa J. (2007). An engineering solution for mesh size effects in the simulation of delamination using cohesive zone models. Eng. Fract. Mech..

[32] Kaw A.K. (2005). Mechanics of Composite Materials.

[33] Song K., Davila J.F., Rose C. Guidelines and parameter selection for the simulation of progressive delamination. Proceedings of the ABAQUS User’s Conference.

[34] Arki S., Ferrero J.F., Marguet S., Redonnet J.M., Aury A. (2019). Strengthening of a curved composite beam by introducing a flat portion. Compos. Struct..

[35] How to Create High Quality STL Files for 3D Prints. https://markforged.com/resources/blog/how-to-create-high-quality-stl-files-for-3d-prints.

